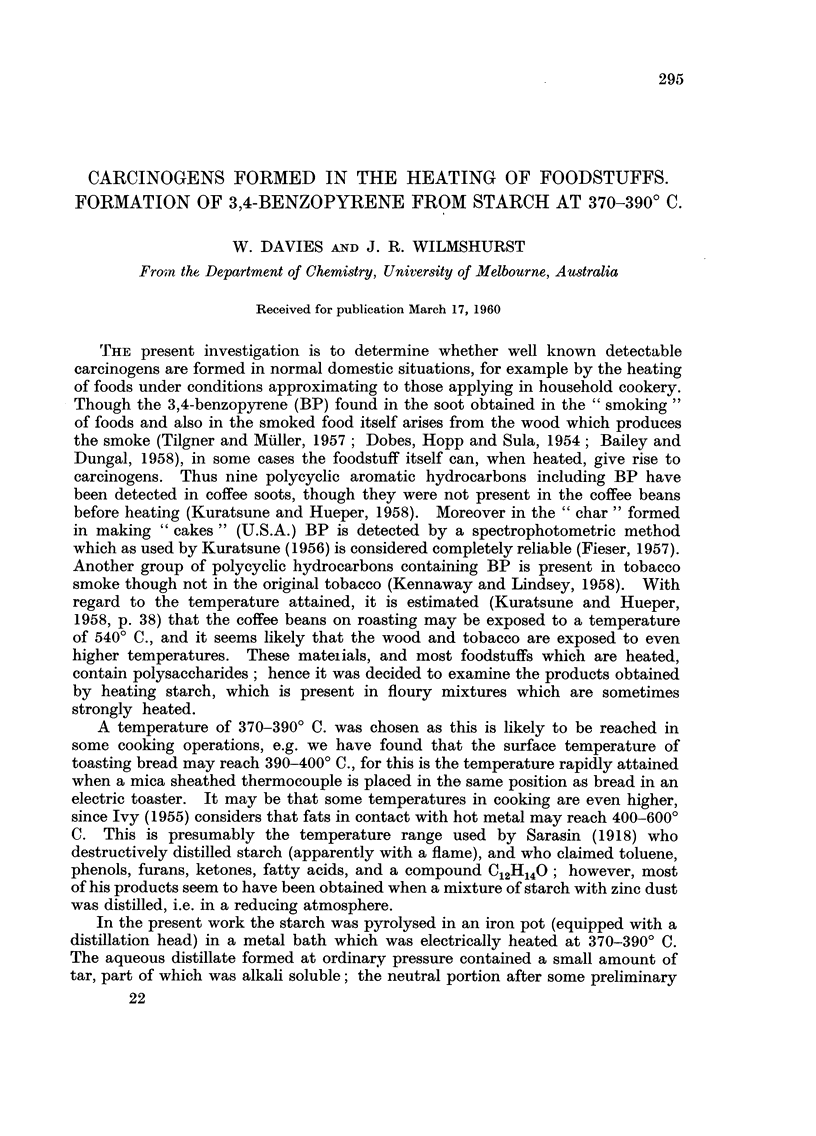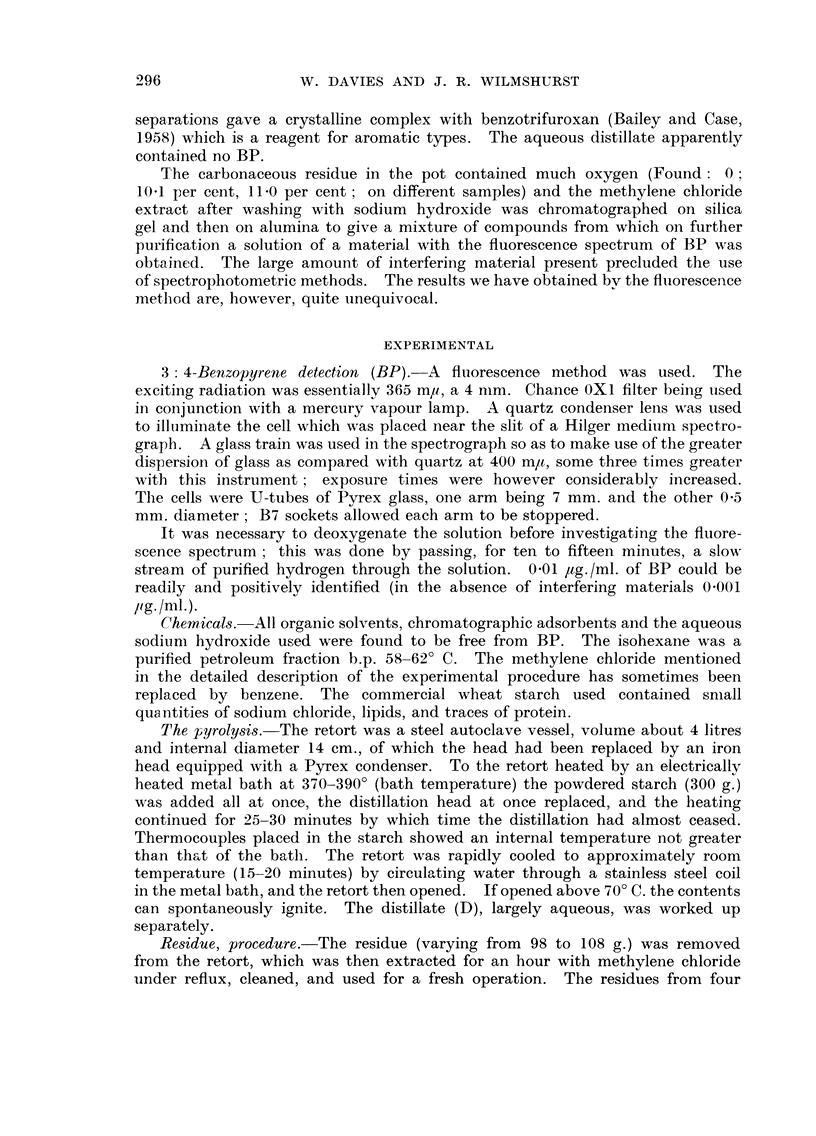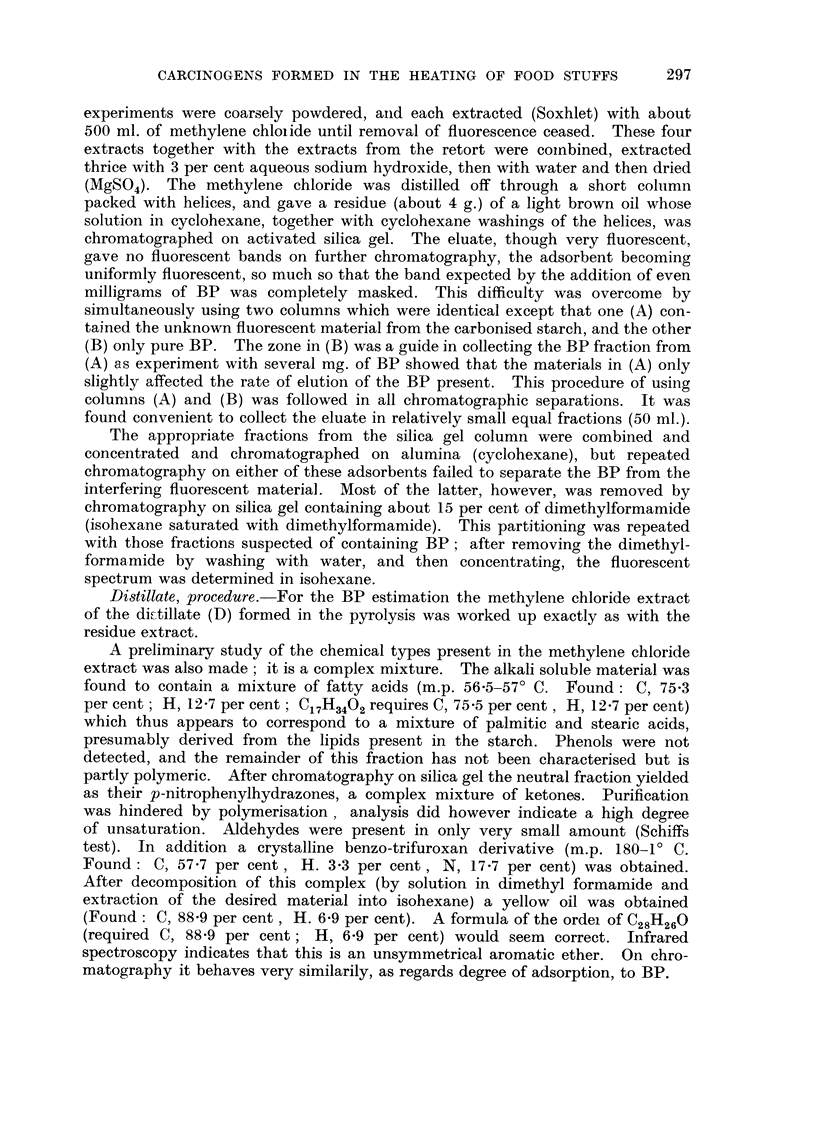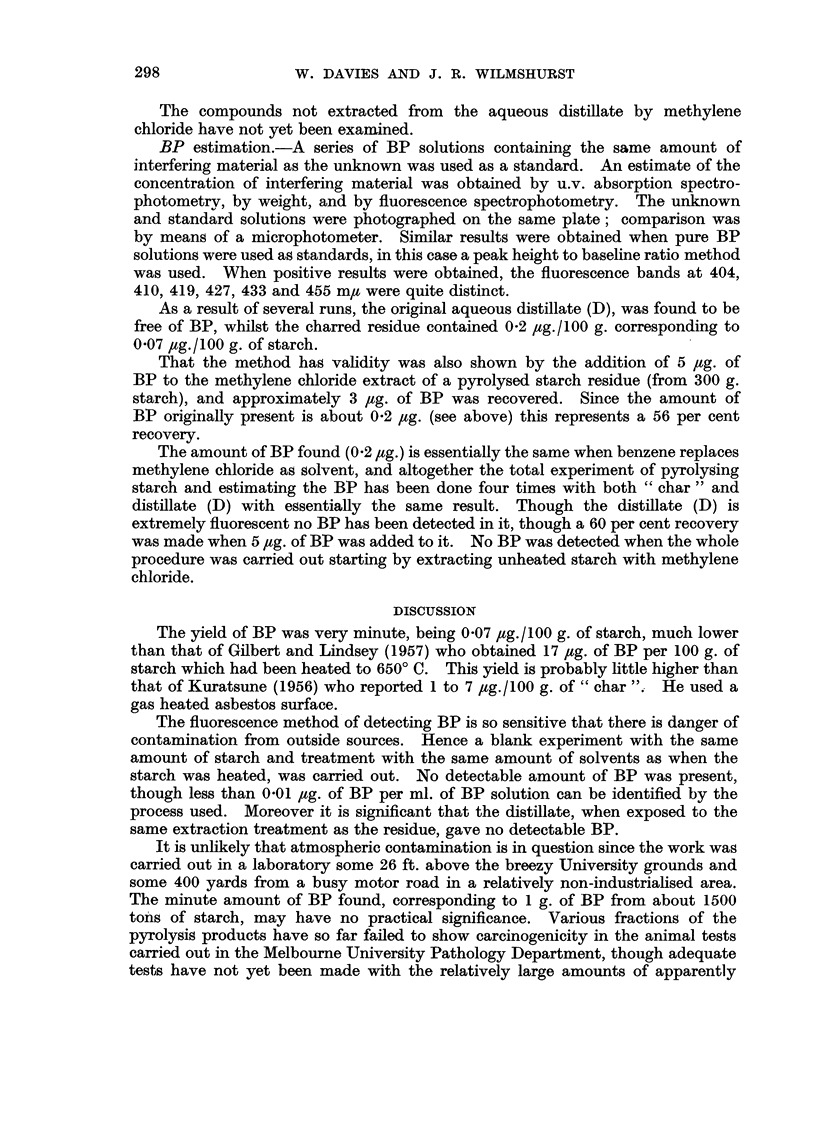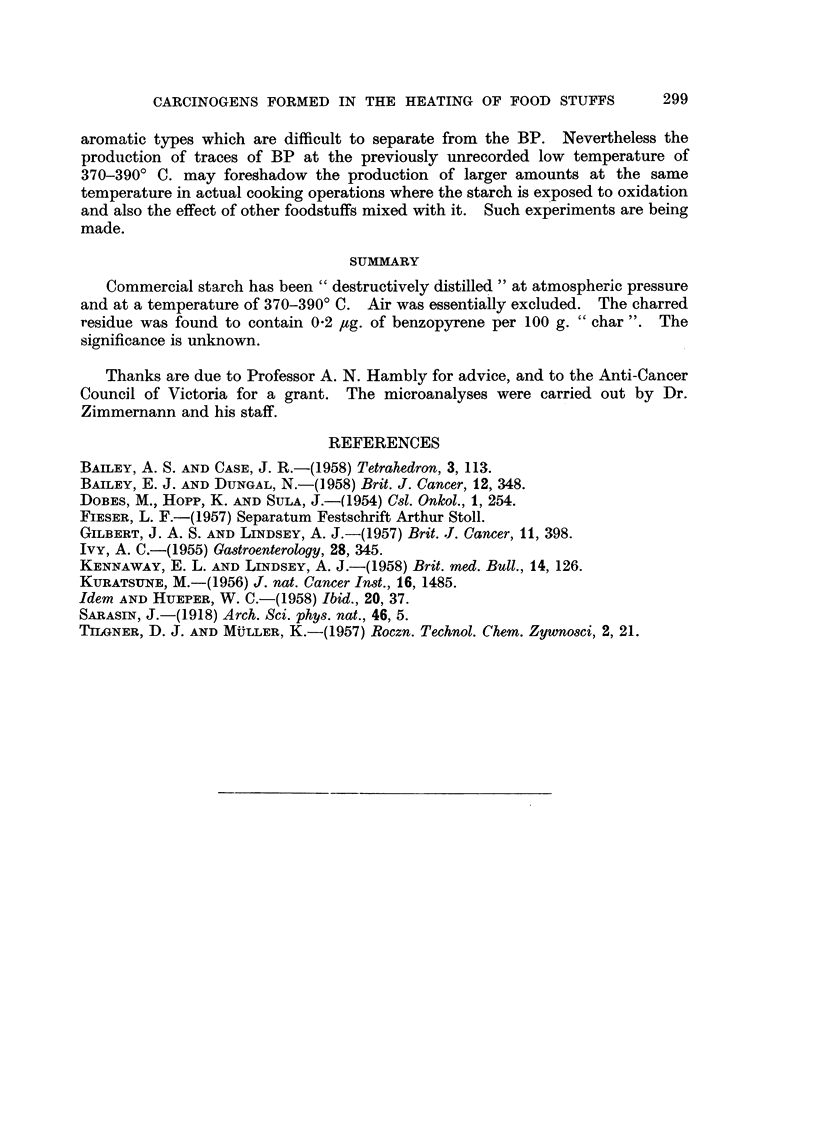# Carcinogens formed in the Heating of Foodstuffs. Formation of 3,4-Benzopyrene from Starch at 370-390° C

**DOI:** 10.1038/bjc.1960.34

**Published:** 1960-06

**Authors:** W. Davies, J. R. Wilmshurst


### CARCINOGENS FORMED IN THE HEATING OF FOODSTUFFS. FORMATION OF 3,4-BENZOPYRENE FROM STARCH AT 370–390° C.

W. DAVIES AND J. R. WILMSHURST

*From the Department of Chemistry, University of Melbourne, Australia*

Received for publication March 17, 1960

THE present investigation is to determine whether well known detectable carcinogens are formed in normal domestic situations, for example by the heating of foods under conditions approximating to those applying in household cookery. Though the 3,4-benzopyrene (BP) found in the soot obtained in the " smoking " of foods and also in the smoked food itself arises from the wood which produces the smoke (Tilgner and Müller, 1957 ; Dobes, Hopp and Sula, 1954 ; Bailey and Dungal, 1958), in some cases the foodstuff itself can, when heated, give rise to carcinogens. Thus nine polycyclic aromatic hydrocarbons including BP have been detected in coffee soots, though they were not present in the coffee beans before heating (Kuratsune and Hueper, 1958). Moreover in the " char " formed in making " cakes " (U.S.A.) BP is detected by a spectrophotometric method which as used by Kuratsune (1956) is considered completely reliable (Fieser, 1957). Another group of polycyclic hydrocarbons containing BP is present in tobacco smoke though not in the original tobacco (Kennaway and Lindsey, 1958). With regard to the temperature attained, it is estimated (Kuratsune and Hueper, 1958, p. 38) that the coffee beans on roasting may be exposed to a temperature of 540° C., and it seems likely that the wood and tobacco are exposed to even higher temperatures. These mateıials, and most foodstuffs which are heated, contain polysaccharides ; hence it was decided to examine the products obtained by heating starch, which is present in floury mixtures which are sometimes strongly heated.

A temperature of 370–390° C. was chosen as this is likely to be reached in some cooking operations, e.g. we have found that the surface temperature of toasting bread may reach 390–400° C., for this is the temperature rapidly attained when a mica sheathed thermocouple is placed in the same position as bread in an electric toaster. It may be that some temperatures in cooking are even higher, since Ivy (1955) considers that fats in contact with hot metal may reach 400–600° C. This is presumably the temperature range used by Sarasin (1918) who destructively distilled starch (apparently with a flame), and who claimed toluene, phenols, furans, ketones, fatty acids, and a compound $C_{12}H_{14}O$ ; however, most of his products seem to have been obtained when a mixture of starch with zinc dust was distilled, i.e. in a reducing atmosphere.

In the present work the starch was pyrolysed in an iron pot (equipped with a distillation head) in a metal bath which was electrically heated at 370–390° C. The aqueous distillate formed at ordinary pressure contained a small amount of tar, part of which was alkali soluble; the neutral portion after some preliminary separations gave a crystalline complex with benzotrifuroxan (Bailey and Case, 1958) which is a reagent for aromatic types. The aqueous distillate apparently contained no BP.

The carbonaceous residue in the pot contained much oxygen (Found : 0 ; 10·1 per cent, 11·0 per cent ; on different samples) and the methylene chloride extract after washing with sodium hydroxide was chromatographed on silica gel and then on alumina to give a mixture of compounds from which on further purification a solution of a material with the fluorescence spectrum of BP was obtained. The large amount of interfering material present precluded the use of spectrophotometric methods. The results we have obtained by the fluorescence method are, however, quite unequivocal.

### EXPERIMENTAL

3 : 4-*Benzopyrene detection* (*BP*).—A fluorescence method was used. The exciting radiation was essentially 365 m$\mu$, a 4 mm. Chance 0X1 filter being used in conjunction with a mercury vapour lamp. A quartz condenser lens was used to illuminate the cell which was placed near the slit of a Hilger medium spectrograph. A glass train was used in the spectrograph so as to make use of the greater dispersion of glass as compared with quartz at 400 m$\mu$, some three times greater with this instrument ; exposure times were however considerably increased. The cells were U-tubes of Pyrex glass, one arm being 7 mm. and the other 0·5 mm. diameter ; B7 sockets allowed each arm to be stoppered.

It was necessary to deoxygenate the solution before investigating the fluorescence spectrum ; this was done by passing, for ten to fifteen minutes, a slow stream of purified hydrogen through the solution. 0·01 $\mu$g./ml. of BP could be readily and positively identified (in the absence of interfering materials 0·001 $\mu$g./ml.).

*Chemicals.*—All organic solvents, chromatographic adsorbents and the aqueous sodium hydroxide used were found to be free from BP. The isohexane was a purified petroleum fraction b.p. 58–62° C. The methylene chloride mentioned in the detailed description of the experimental procedure has sometimes been replaced by benzene. The commercial wheat starch used contained small quantities of sodium chloride, lipids, and traces of protein.

*The pyrolysis.*—The retort was a steel autoclave vessel, volume about 4 litres and internal diameter 14 cm., of which the head had been replaced by an iron head equipped with a Pyrex condenser. To the retort heated by an electrically heated metal bath at 370–390° (bath temperature) the powdered starch (300 g.) was added all at once, the distillation head at once replaced, and the heating continued for 25–30 minutes by which time the distillation had almost ceased. Thermocouples placed in the starch showed an internal temperature not greater than that of the bath. The retort was rapidly cooled to approximately room temperature (15–20 minutes) by circulating water through a stainless steel coil in the metal bath, and the retort then opened. If opened above 70° C. the contents can spontaneously ignite. The distillate (D), largely aqueous, was worked up separately.

*Residue, procedure.*—The residue (varying from 98 to 108 g.) was removed from the retort, which was then extracted for an hour with methylene chloride under reflux, cleaned, and used for a fresh operation. The residues from four experiments were coarsely powdered, and each extracted (Soxhlet) with about 500 ml. of methylene chloride until removal of fluorescence ceased. These four extracts together with the extracts from the retort were combined, extracted thrice with 3 per cent aqueous sodium hydroxide, then with water and then dried ($MgSO_4$). The methylene chloride was distilled off through a short column packed with helices, and gave a residue (about 4 g.) of a light brown oil whose solution in cyclohexane, together with cyclohexane washings of the helices, was chromatographed on activated silica gel. The eluate, though very fluorescent, gave no fluorescent bands on further chromatography, the adsorbent becoming uniformly fluorescent, so much so that the band expected by the addition of even milligrams of BP was completely masked. This difficulty was overcome by simultaneously using two columns which were identical except that one (A) contained the unknown fluorescent material from the carbonised starch, and the other (B) only pure BP. The zone in (B) was a guide in collecting the BP fraction from (A) as experiment with several mg. of BP showed that the materials in (A) only slightly affected the rate of elution of the BP present. This procedure of using columns (A) and (B) was followed in all chromatographic separations. It was found convenient to collect the eluate in relatively small equal fractions (50 ml.).

The appropriate fractions from the silica gel column were combined and concentrated and chromatographed on alumina (cyclohexane), but repeated chromatography on either of these adsorbents failed to separate the BP from the interfering fluorescent material. Most of the latter, however, was removed by chromatography on silica gel containing about 15 per cent of dimethylformamide (isohexane saturated with dimethylformamide). This partitioning was repeated with those fractions suspected of containing BP ; after removing the dimethylformamide by washing with water, and then concentrating, the fluorescent spectrum was determined in isohexane.

*Distillate, procedure.*—For the BP estimation the methylene chloride extract of the distillate (D) formed in the pyrolysis was worked up exactly as with the residue extract.

A preliminary study of the chemical types present in the methylene chloride extract was also made ; it is a complex mixture. The alkali soluble material was found to contain a mixture of fatty acids (m.p. 56·5–57° C. Found : C, 75·3 per cent ; H, 12·7 per cent ; $C_{17}H_{34}O_2$ requires C, 75·5 per cent , H, 12·7 per cent) which thus appears to correspond to a mixture of palmitic and stearic acids, presumably derived from the lipids present in the starch. Phenols were not detected, and the remainder of this fraction has not been characterised but is partly polymeric. After chromatography on silica gel the neutral fraction yielded as their *p*-nitrophenylhydrazones, a complex mixture of ketones. Purification was hindered by polymerisation , analysis did however indicate a high degree of unsaturation. Aldehydes were present in only very small amount (Schiffs test). In addition a crystalline benzo-trifuroxan derivative (m.p. 180–1° C. Found : C, 57·7 per cent , H. 3·3 per cent , N, 17·7 per cent) was obtained. After decomposition of this complex (by solution in dimethyl formamide and extraction of the desired material into isohexane) a yellow oil was obtained (Found : C, 88·9 per cent , H. 6·9 per cent). A formula of the order of $C_{28}H_{26}O$ (required C, 88·9 per cent ; H, 6·9 per cent) would seem correct. Infrared spectroscopy indicates that this is an unsymmetrical aromatic ether. On chromatography it behaves very similarily, as regards degree of adsorption, to BP.

The compounds not extracted from the aqueous distillate by methylene chloride have not yet been examined.

*BP* estimation.—A series of BP solutions containing the same amount of interfering material as the unknown was used as a standard. An estimate of the concentration of interfering material was obtained by u.v. absorption spectrophotometry, by weight, and by fluorescence spectrophotometry. The unknown and standard solutions were photographed on the same plate ; comparison was by means of a microphotometer. Similar results were obtained when pure BP solutions were used as standards, in this case a peak height to baseline ratio method was used. When positive results were obtained, the fluorescence bands at 404, 410, 419, 427, 433 and 455 m$\mu$ were quite distinct.

As a result of several runs, the original aqueous distillate (D), was found to be free of BP, whilst the charred residue contained 0·2 $\mu$g./100 g. corresponding to 0·07 $\mu$g./100 g. of starch.

That the method has validity was also shown by the addition of 5 $\mu$g. of BP to the methylene chloride extract of a pyrolysed starch residue (from 300 g. starch), and approximately 3 $\mu$g. of BP was recovered. Since the amount of BP originally present is about 0·2 $\mu$g. (see above) this represents a 56 per cent recovery.

The amount of BP found (0·2 $\mu$g.) is essentially the same when benzene replaces methylene chloride as solvent, and altogether the total experiment of pyrolysing starch and estimating the BP has been done four times with both " char " and distillate (D) with essentially the same result. Though the distillate (D) is extremely fluorescent no BP has been detected in it, though a 60 per cent recovery was made when 5 $\mu$g. of BP was added to it. No BP was detected when the whole procedure was carried out starting by extracting unheated starch with methylene chloride.

### DISCUSSION

The yield of BP was very minute, being 0·07 $\mu$g./100 g. of starch, much lower than that of Gilbert and Lindsey (1957) who obtained 17 $\mu$g. of BP per 100 g. of starch which had been heated to 650° C. This yield is probably little higher than that of Kuratsune (1956) who reported 1 to 7 $\mu$g./100 g. of " char ". He used a gas heated asbestos surface.

The fluorescence method of detecting BP is so sensitive that there is danger of contamination from outside sources. Hence a blank experiment with the same amount of starch and treatment with the same amount of solvents as when the starch was heated, was carried out. No detectable amount of BP was present, though less than 0·01 $\mu$g. of BP per ml. of BP solution can be identified by the process used. Moreover it is significant that the distillate, when exposed to the same extraction treatment as the residue, gave no detectable BP.

It is unlikely that atmospheric contamination is in question since the work was carried out in a laboratory some 26 ft. above the breezy University grounds and some 400 yards from a busy motor road in a relatively non-industrialised area. The minute amount of BP found, corresponding to 1 g. of BP from about 1500 tons of starch, may have no practical significance. Various fractions of the pyrolysis products have so far failed to show carcinogenicity in the animal tests carried out in the Melbourne University Pathology Department, though adequate tests have not yet been made with the relatively large amounts of apparently aromatic types which are difficult to separate from the BP. Nevertheless the production of traces of BP at the previously unrecorded low temperature of 370–390° C. may foreshadow the production of larger amounts at the same temperature in actual cooking operations where the starch is exposed to oxidation and also the effect of other foodstuffs mixed with it. Such experiments are being made.

SUMMARY

Commercial starch has been " destructively distilled " at atmospheric pressure and at a temperature of 370–390° C. Air was essentially excluded. The charred residue was found to contain 0·2 μg. of benzopyrene per 100 g. " char ". The significance is unknown.

Thanks are due to Professor A. N. Hambly for advice, and to the Anti-Cancer Council of Victoria for a grant. The microanalyses were carried out by Dr. Zimmernann and his staff.

### REFERENCES

Bailey, A. S. and Case, J. R.—(1958) *Tetrahedron*, **3**, 113.
Bailey, E. J. and Dungal, N.—(1958) *Brit. J. Cancer*, **12**, 348.
Dobes, M., Hopp, K. and Sula, J.—(1954) *Csl. Onkol.*, **1**, 254.
Fieser, L. F.—(1957) Separatum Festschrift Arthur Stoll.
Gilbert, J. A. S. and Lindsey, A. J.—(1957) *Brit. J. Cancer*, **11**, 398.
Ivy, A. C.—(1955) *Gastroenterology*, **28**, 345.
Kennaway, E. L. and Lindsey, A. J.—(1958) *Brit. med. Bull.*, **14**, 126.
Kuratsune, M.—(1956) *J. nat. Cancer Inst.*, **16**, 1485.
*Idem* and Hueper, W. C.—(1958) *Ibid.*, **20**, 37.
Sarasin, J.—(1918) *Arch. Sci. phys. nat.*, **46**, 5.
Tilgner, D. J. and Müller, K.—(1957) *Roczn. Technol. Chem. Zywnosci*, **2**, 21.